# Identification of RNA-Binding Protein Targets with HyperTRIBE in *Saccharomyces cerevisiae*

**DOI:** 10.3390/ijms24109033

**Published:** 2023-05-20

**Authors:** Weilan Piao, Chong Li, Pengkun Sun, Miaomiao Yang, Yansong Ding, Wei Song, Yunxiao Jia, Liqun Yu, Yanming Lu, Hua Jin

**Affiliations:** Key Laboratory of Molecular Medicine and Biotherapy, School of Life Science, Beijing Institute of Technology, No. 5 South Zhongguancun Street, Beijing 100081, China; weilanpiao@bit.edu.cn (W.P.);

**Keywords:** HyperTRIBE, RNA-binding protein (RBP), yeast, KHD1, BFR1

## Abstract

As a master regulator in cells, RNA-binding protein (RBP) plays critical roles in organismal development, metabolism and various diseases. It regulates gene expression at various levels mostly by specific recognition of target RNA. The traditional CLIP-seq method to detect transcriptome-wide RNA targets of RBP is less efficient in yeast due to the low UV transmissivity of their cell walls. Here, we established an efficient HyperTRIBE (Targets of RNA-binding proteins Identified By Editing) in yeast, by fusing an RBP to the hyper-active catalytic domain of human RNA editing enzyme ADAR2 and expressing the fusion protein in yeast cells. The target transcripts of RBP were marked with new RNA editing events and identified by high-throughput sequencing. We successfully applied HyperTRIBE to identifying the RNA targets of two yeast RBPs, KHD1 and BFR1. The antibody-free HyperTRIBE has competitive advantages including a low background, high sensitivity and reproducibility, as well as a simple library preparation procedure, providing a reliable strategy for RBP target identification in *Saccharomyces cerevisiae*.

## 1. Introduction

RNA-binding protein (RBP) specifically interacts with RNA in cells and is widely involved in the regulation of gene expression [1]. RBP–RNA interactions lay the foundation of cellular functions; thus, the investigation of the RBP–RNA interaction network is of great significance for understanding different cellular processes and the fundamental roles of RBP in these processes. Hundreds of RBPs have been revealed in mammals [2,3], and multiple RBPs usually cooperate in a complex while some proteins in the complex are indirectly associated with RNA, which gives rise to difficulties in the detection of RNA–protein interactions [4,5].

The importance of RNA–protein interactions has facilitated rapid development of profiling methods for RBP targets at the transcriptome level. These methods include RNA-immunoprecipitation sequencing (RIP-seq), high-throughput sequencing of RNA isolated by crosslinking immunoprecipitation (HITS-CLIP) and its variants, Targets of RNA-binding proteins Identified By Editing (TRIBE), HyperTRIBE, RNA tagging, and Surveying Targets by APOBEC-Mediated Profiling (STAMP) [6,7,8,9,10,11,12]. Of the above, HITS-CLIP (CLIP-seq) has been widely used as a powerful method for detecting RBP-binding sites on RNA at a single-nucleotide resolution [9]. The original HITS-CLIP method relies on high-quality antibodies and large quantities of cells with low UV-crosslinking efficiency; however, its variants have greatly improved the efficiency [10,11,12].

In recent years, three antibody-independent methods have been developed, all by fusing an RNA modification enzyme to an RBP to mark RBP targets, thereby providing alternative strategies for the study of RBPs. TRIBE and RNA tagging were developed in late 2015 to early 2016 and STAMP was established in 2021 [6,7,8]. In TRIBE, an RBP is fused with the catalytic domain of adenosine deaminase ADAR (ADARcd) to produce a fusion protein [6]. When the RBP in the fusion protein binds to target RNAs, ADARcd edits adjacent adenosine (A) into inosine (I). During high-throughput sequencing, inosine (I) is read as guanosine (G), thereby identifying the target transcripts of the RBP. In its improved version HyperTRIBE, the ADARcd possesses an amino acid substitution, making it able to work with higher detection sensitivity and less bias during binding site identification when compared with TRIBE. In other words, it determines targets with lower sequencing depth and lower false-negative rates compared with TRIBE [13,14,15]. TRIBE has been employed to flies [6,13,15,16,17], mammals [13,18], malaria parasites [19] and plants [20]. In RNA tagging [8], an RBP is fused with poly(U) polymerase (PUP-2) [21] in yeasts. PUP-2, originated from *C. elegans,* adds a poly(U) tail to the 3′ end of target RNA and the U-tailed mRNA is then identified by paired-end sequencing. As poly(U) polymerase modifies the 3′ end of the mRNA poly(A) tail, RNA tagging is supposed to work better for RBPs that bind to the 3′ UTR since the region is close to 3′ terminus. STAMP [7] in mammals is designed to fuse the RNA cytosine deaminase APOBEC1 [22] from *Rattus norvegicus* with an RBP and then determine cytosine to uridine transitions in target mRNA by sequencing. Current research has indicated that STAMP works well in human cells but it is hard to applied in *Drosophila* cells [23]. Furthermore, it is uncertain that RNA modification enzymes have activity in other untested species. Therefore, utilization of these methods in other species calls for further verification and optimization.

Yeasts are remarkable eukaryotic model organisms for basic studies and bioengineering [24]. To date, approximately 120 yeast proteins have been found to be associated with mRNA [25], many of which are conserved in higher organisms. The typical RBP KHD1 is a *S. cerevisiae* homolog of mammalian heterogeneous nuclear ribonucleoprotein K (hnRNP K) [26], and includes three conserved K homology (KH) domains. Under suitable environments, yeast cells undergo asexual reproduction mainly by budding. Genome screening has showed that KHD1 interacts with approximately 50% of bud localization mRNAs [27,28], most of which encode membrane or secretory proteins [29]. In addition, KHD1 has been reported to have distinct effects on different target genes. For example, KHD1 inhibits translation of FLO11 mRNA [30] while it stabilizes MTL1 mRNA [28,31]. During asymmetric cell division [32,33,34], KHD1 inhibits ASH1 mRNA translation and participates in the accumulation of ASH1 protein in the growing bud, thereby impeding mating type conversion in daughter cells [35].

Another multi-functional RBP, BFR1, is involved in the uneven distribution of mRNA during yeast budding and responsible for target mRNA localization in the bud tip [36]. BFR1 is localized to the endoplasmic reticulum [25,37] and is a component of polyribosome-associated mRNP complexes [38]. It is also located in late P-bodies and is very important for the delayed entry of specific mRNAs into the P-body, such as VNX1 and TDP1 mRNAs [39]. It has been proposed that BFR1 protein may mediate the transformation of target mRNA from translation to degradation under stress conditions such as starvation, resulted in the accumulation of BFR1 protein in P-bodies [39].

Because the rigid cell wall leads to less efficiency of UV cross-linking for CLIP in organisms like plants, fungi and bacteria, TRIBE could be a good choice there. In addition, multiple available tools for RBP target examination in one species will be conducive to the confident identification of RBP targets as well as to studying several cooperative RBPs in a large complex simultaneously. Thus, we pioneered HyperTRIBE in *S. cerevisiae* and investigated the target transcripts of two RBPs, KHD1 and BFR1. Our results showed that HyperTRIBE performed well with a low background, good sensitivity and reproducibility. Additionally, combined with the identification of differentially expressed genes (DEGs) after depletion of KHD1 or BFR1, we identified key targets of KHD1 and BFR1 and their functions. Our work established an effective method to identify RBP targets in yeast with a simple procedure and will be beneficial for the field.

## 2. Results

### 2.1. KHD1-HyperTRIBE Successfully Identified KHD1 Targets

**Figure 1:**
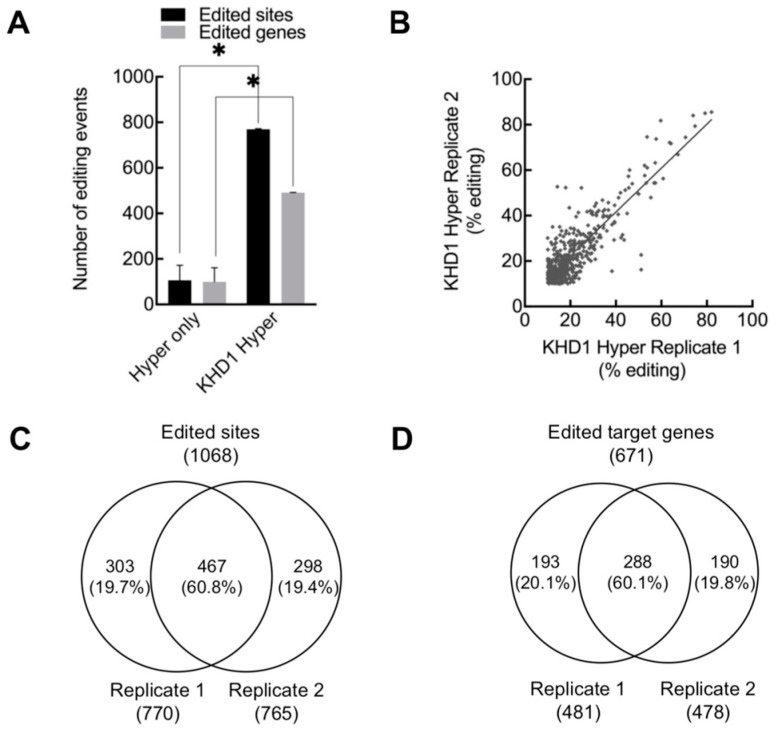
KHD1-HyperTRIBE identified target RNAs of KHD1 in yeast with reproducibility. (**A**) The edited sites and edited genes detected in KHD1-HyperTRIBE were significantly higher than those detected in Hyper-only. KHD1-HyperTRIBE detected ~770 edited sites and ~500 target genes on average (Editing ≥ 10%, read ≥ 20). *N* = 2; mean + SEM; * *p* < 0.05, paired one-tailed Student’s *t* test. (**B**) The editing degree of detected sites in two biological replicates was similar (R^2^ = 0.74). (**C**,**D**) About 60% of edited sites and edited genes were reproducibly detected in the two biological replicates as showed in the Venn diagrams.

KHD1 protein possesses three KH domains, which are one of the well-known classical RNA-binding motifs (Figure S1A). KHD1 was reported to inhibit the translation initiation of hundreds of mRNAs in the process of mRNA transfer to specific cell loci, indicating that it has hundreds of potential mRNA targets [28]. To identify the targets of KHD1 in yeast, KHD1 was fused with the hyper-active catalytic domain of human ADAR2 E488Q (hADAR2cd) (Figure S1B) because hADAR2cd had been used in mammalian HyperTRIBE [13] and was confirmed to have activity in yeast cells [40]. The protein expression of hADAR2cd (Hyper-only) and KHD1-hADAR2cd (KHD1 Hyper) in *S. cerevisiae* was validated by Western blotting (Figure S1C). Very few A-to-G editing events were detected in the negative control Hyper-only, while 770 edited sites and 492 target genes on average were detected in KHD1-HyperTRIBE, indicating that HyperTRIBE works well in yeast cells (Figure 1A). The edited sites in KHD1-HyperTRIBE were mainly located in the protein-coding region and 3′ UTR but not in the 5′ UTR (Figure S2A). In addition, the base editing in KHD1-HyperTRIBE was mostly restricted to A-to-G, and no other types of base editing were significantly enriched in KHD1-HyperTRIBE compared to Hyper-only (Figure S2C). This observation indicated that A-to-G editing events indeed resulted from the KHD1-fused hADAR2cd activity and not from non-specific or other enzymatic activities.

The editing events in Hyper-only and KHD1-HyperTRIBE were highly distinguishable since only 5.3% of edited sites were common between them and the editing percentages in KHD1-HyperTRIBE were significantly higher than those in Hyper-only for the common edited sites, revealing that HyperTRIBE can specifically identify target transcripts (Figure S3A,B). The editing events in KHD1-HyperTRIBE were highly reproducible both in frequencies and positions (Figure 1B,C and Table S1), revealing that the method specifically identified target transcripts. With the threshold of a read number of 20 and editing percentage of 10% (r20e10), 288 (60%) genes were marked in the two biological replicates and defined as high-confidence targets. A total of 671 target genes was detected at least once in the two biological replicates, accounting for 11% of all *S. cerevisiae* genes (Figure 1D and Table S2).

**Figure 2:**
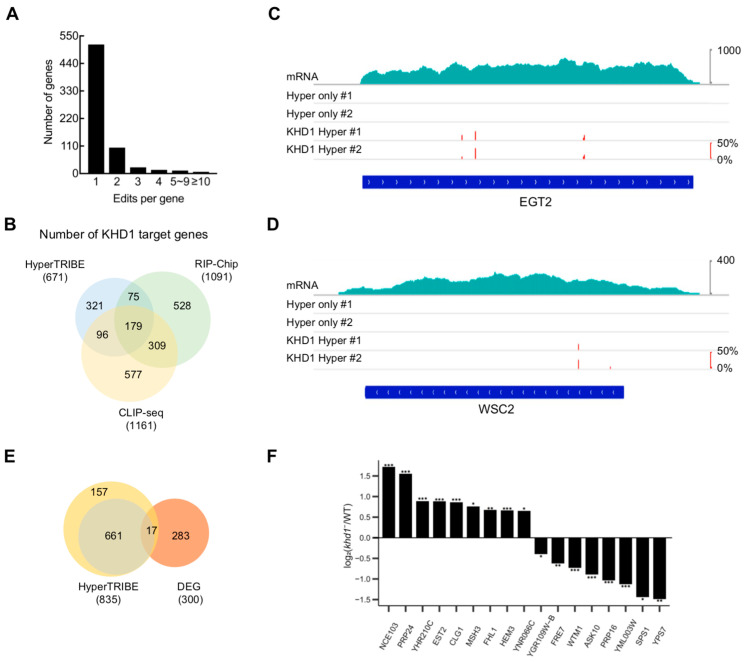
KHD1-HyperTRIBE targets overlap well with the KHD1 targets detected by other methods. (**A**) The frequency histogram of edited sites per gene shows that 76% of target genes of KHD1 were edited once in KHD1-HyperTRIBE. (**B**) The comparison of targets detected in KHD1-HyperTRIBE, RIP-Chip (SGD) [28] and CLIP-seq (POSTAR3) [30] indicates that 350 (52%) of the HyperTRIBE target genes have been detected by the other two methods. The KHD1 HyperTRIBE results have significant overlap with those of RIP-Chip and CLIP-seq. *p* < 0.0001, Fisher’s Exact Test. (**C**,**D**) The IGV view of well-known KHD1 targets shows that KHD1-HyperTRIBE specifically edited target RNAs with high editing efficiency and reproducibility. The edited sites and editing percentages of EGT2 and WSC2 are shown as short red bars, and the heights represent the editing percentages of loci. (**E**) As shown in the Venn diagram, seventeen genes overlapped between KHD1-HyperTRIBE targets and differentially expressed genes (DEG) in *khd1*-depleted (*khd^−^*) yeast observed by microarray analysis of mRNA (NCBI). *p* < 0.0001. The threshold of the yellow circle is r10e10, and the threshold of the green circle is r20e10. The cutoff of the DEGs is *p* < 0.1 and |log (*khd1^−^*/WT)| > 0.2. (**F**) For the overlapping 17 genes, the mRNA level changes in *khd1^−^* yeasts compared with wild type (WT) are shown. The bar chart shows log_2_(*khd1^−^*/WT). *N* = 3, * *p* < 0.1, ** *p* < 0.05, *** *p* < 0.01, paired one-tailed Student’s *t* test. SGD: Saccharomyces Genome Database, IGV: Integrative Genomics Viewer, DEG: differentially expressed gene.

The frequency histogram of edited sites per KHD1 target gene showed that 76% of target genes possessed a single edited site, and 24% had more than one edited site (Figure 2A). When the KHD1-HyperTRIBE target genes were compared with the results of RNA immunoprecipitation-Chip (RIP-Chip) [28] and CLIP-seq [30], half of the HyperTRIBE targets were detected at least once in the other methods and this overlapping ratio was similar to that of the other two methods (Figure 2B). Interestingly, the well-known bud tip localization mRNAs like MTL1, WSC2, EGT2 and IST2 [29] were identified as KHD1 targets in KHD1-HyperTRIBE (Figure 2C,D and Figure S4). As mentioned before, a total of 671 target genes was detected at least once in the two replication experiments of KHD1-HyperTRIBE with the threshold of a read number of 20 and editing percentage of 10% (r20e10). When the threshold was set as r10e10, a total of 835 targets was identified at least once (Figure 2E). In addition, we obtained the results of mRNA expression profiling by microarray in wild-type and *khd1*-depleted yeast strains from NCBI, and acquired 300 differentially expressed genes (*p* < 0.1 and |log (*khd1^−^*/WT)| > 0.2). Among them, 17 genes overlapped with the 835 KHD1-HyperTRIBE targets (Figure 2E,F), while up-regulated and down-regulated genes had similar proportions in these 17 genes (Figure 2F), suggesting that KHD1 binding has different impacts on target mRNA levels.

**Figure 3:**
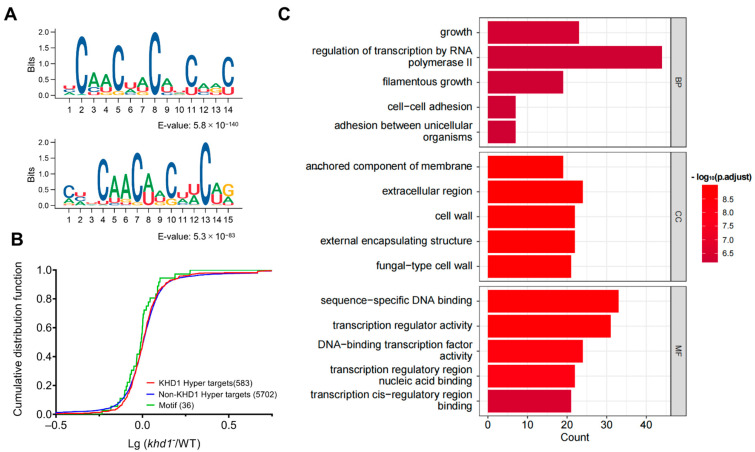
The targets of KHD1-HyperTRIBE have specific motifs and their functions are enriched in filamentous growth and cell adhesion. (**A**) The motifs identified by KHD1-HyperTRIBE included CAACAA and CAUCAU, which are consistent with the results of the published KH domain structure and KHD1 CLIP-seq analysis. The motif analysis was conducted using the XSTREME (up) and MEME (down) algorithms and the ±100 nt sequences of the high confident KHD1-HyperTRIBE sites. (**B**) Cumulative distributions of the log mRNA-abundance-ratio of *khd1* knockout yeast (*khd1^−^*) to its wild-type counterpart (WT) from KHD1-TRIBE target group (red line, *n* = 583), non-target group (blue line, *n* = 5702) and the target group with motif (CAACAA) (green line, *n* = 36). There were no significantly different distributions among the three groups (all the *p* > 0.1). *p*-value was calculated by ordinary one-way ANOVA between each pair of groups. (**C**) GO term analysis indicated that the roles of KHD1 target genes are enriched in transcriptional regulation, cell growth, filamentous growth and cell adhesion, which correlate well with the reported *khd1*-knockout yeast phenotype. BP: biological process, CC: cellular component, MF: molecular function.

To determine the consensus sequence in KHD1-binding sites, we next carried out MEME motif analysis. It was shown that ~50% of RBP-TRIBE edited sites generally appear within 100 nt of CLIP-seq sites [6]. Thus, we conducted motif analysis using the regions 100 nt upstream and 100 nt downstream from the KHD1-HyperTRIBE edited sites. The analysis revealed that YCAACAA and YCAUCAU (Y represents a pyrimidine, U or C) motifs were enriched in the KHD1-binding regions (Figure 3A), consistent with structural studies of the third KH domain of Nova which specifically interacts with the internal CA in a YCAY (Y represents a pyrimidine, U or C) motif [41] and similar to the previously published CLIP-seq data [30]. To explore whether the binding of KHD1 protein to its target transcripts affects target mRNA levels, the steady state mRNA abundance changes after knocking out *khd1* were compared among three different groups of genes: KHD1-HyperTRIBE targets, KHD1-HyperTRIBE targets with the identified motif (CAACAA) and non-targets (Figure 3B). Cumulative frequency distributions of the mRNA level changes showed that there were no significantly different distributions among three groups, suggesting that KHD1 is not involved in general mRNA stability control (Figure 3B). 

Gene ontology (GO) term analysis showed that the target genes of KHD1 are related to filamentous growth, cell growth and adhesion (Figure 3C). Interestingly, it was reported that *khd1*-knockout yeast has increased filamentous growth, and ability to adhere together and to agar plate [30], indicating that KHD1-HyperTRIBE convincingly captured KHD1 targets. It also suggested that the binding of KHD1 to its target transcripts controls the target expression in certain ways thereby inhibiting filamentous growth and cell adhesion. The HyperTRIBE results provide a basis for further functional and mechanistic studies of KHD1.

### 2.2. The BFR1-HyperTRIBE Exhibits BFR1-Determined Editing Activity

**Figure 4:**
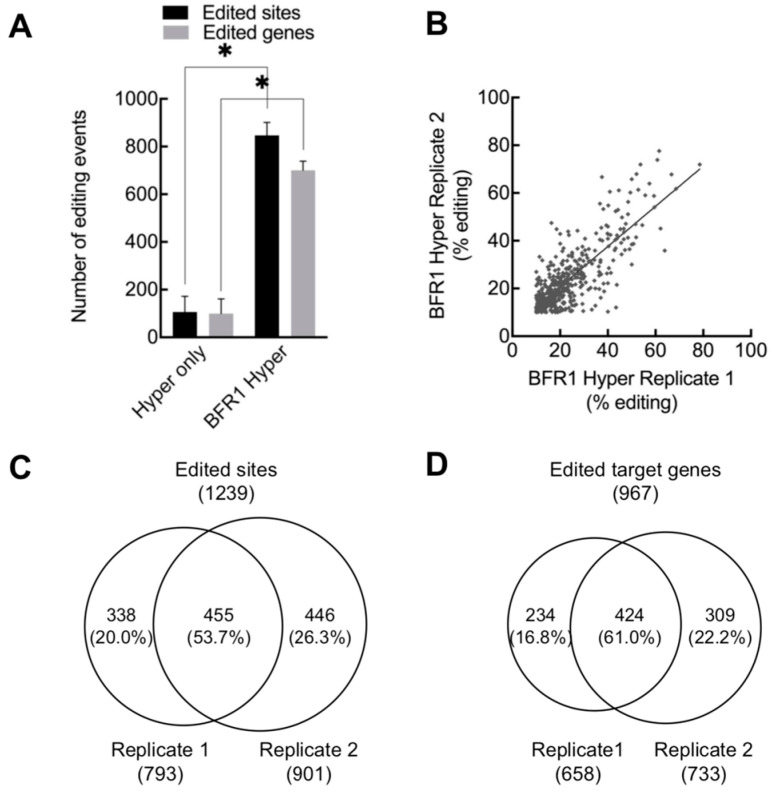
BFR1-HyperTRIBE successfully edited the target RNAs of BFR1 with high reproducibility. (**A**) BFR1-HyperTRIBE generated many more edited sites and edited genes than Hyper-only, indicating that BFR1-HyperTRIBE efficiently identified BFR1 targets (Read ≥ 20, editing ≥ 10%). The editing events are averaged over two replicate experiments. *N* = 2, +SEM; * *p* = 0.05, paired one-tailed Student’s *t* test. (**B**) The editing percentages of detected sites in two biological replicates were similar (R^2^ = 0.63). (**C**,**D**) Venn diagram shows that ~60% of edited sites and edited genes were reproducibly detected in the two replicates.

To establish HyperTRIBE in yeast, we further tested the method by using another multi-functional RBP: BFR1. Even though BFR1 includes only a Structural Maintenance of Chromosomes (Smc) domain and lacks any classical RNA-binding domains (Figure S1A), it is well known to be widely involved in mRNA metabolism. The HyperTRIBE results showed that in *S. cerevisiae* cells, the proteins of hADAR2cd (Hyper-only) and BFR1-hADAR2cd (BFR1-Hyper) were expressed well after induction (Figure S1C). Then, the numbers of edited sites and edited genes were counted in the Hyper-only and BFR1-Hyper RNA-sequencing results. The 106 edited sites and 100 edited genes were detected in the negative control Hyper-only group, while 847 edited sites and 700 edited genes were observed in the BFR1-Hyper group on average; the ratio of signal to noise was more than seven (Figure 4A). The edited sites were observed in the protein-coding region and 3′ UTR but not in the 5′ UTR (Figure S2B). A significantly high level of A-to-G editing but no other types of noticeable editing were observed in BFR1-Hyper samples when compared with Hyper-only samples (Figure S2C). The identified edited sites between Hyper-only and BFR1-HyperTRIBE were compared with many more edited sites and editing percentages in BFR1-HyperTRIBE, revealing that HyperTRIBE can specifically identify target transcripts (Figure S3C,D). In addition, the editing events were repeatable in their editing percentages, positions and target genes (Figure 4B–D and Table S3), and ~61% of target genes were reproducibly detected (Figure 4D and Table S4), indicating that the target identification in BFR1-HyperTRIBE was highly specific.

**Figure 5:**
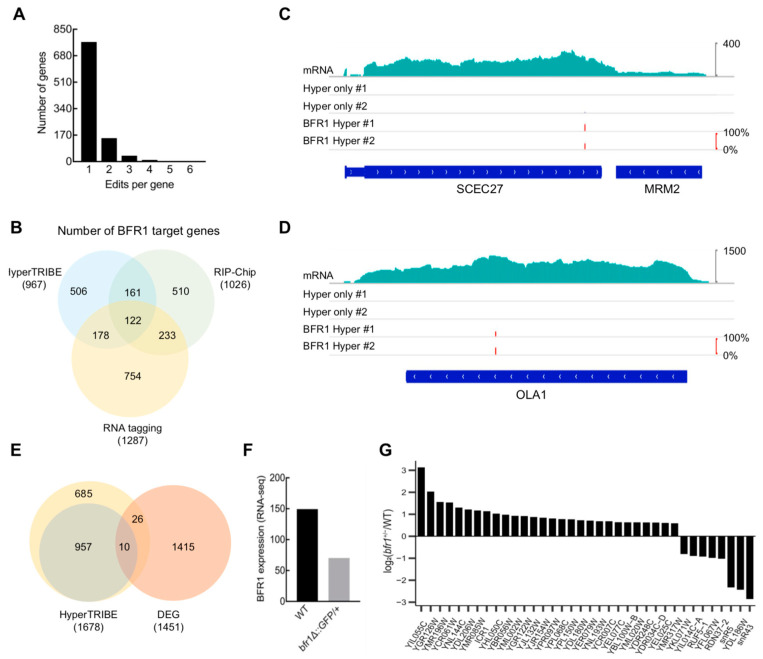
BFR1-HyperTRIBE targets overlap well with the BFR1 targets detected by other methods. (**A**) The frequency histogram of edited sites per gene shows that most target genes of BFR1 were edited once in BFR1-HyperTRIBE. (**B**) The comparison of targets detected in BFR1-HyperTRIBE, RIP-Chip [42] and RNA tagging [8] indicates that half of the HyperTRIBE targets have been detected by the other two methods. The BFR1-HyperTRIBE results have significant overlap with those of RIP-Chip and RNA tagging. *p* < 0.0001, Fisher’s Exact Test. (**C**,**D**) The IGV view of two representative BFR1 targets detected in BFR1-HyperTRIBE. The edited sites and editing percentages of SCEC27 and OLA1 are shown as short red bars, and the heights represent the editing percentages of loci. (**E**) Thirty six genes overlapped between BFR1-HyperTRIBE and DEGs of *bfr1*^+/−^ (*bfr1Δ::GFP/+*) yeast observed by RNA-seq (NCBI). The cutoff for the DEGs is FPKM_avg_ ≥ 2 and FPKM ratio multiples greater than 1.5. The HyperTRIBE threshold is r10e10 for the yellow circle and r20e10 for the green circle. (**F**) The BFR1 mRNA expression in the *bfr1*^+/−^ yeast strain was about half that of WT. (**G**) The bar chart shows log_2_(*bfr1*^+/−^/WT) of 36 overlapping genes. DEG: differentially expressed gene.

The frequency histogram of edited sites per BFR1 target gene showed that most target genes have only one edited site (Figure 5A). When the 967 target genes (r20e10), which were detected at least once between the two BFR1-HyperTRIBE experiments, were compared with the published BFR1-RNA Tagging data [8] and RIP-Chip data [42], about half of the BFR1-HyperTRIBE targets were detected at least once by the other two methods (Figure 5B). The representative BFR1-HyperTRIBE targets SCEC27 and OLA1 were identified in two independent experiments with high editing percentages (Figure 5C,D); SCEC27 is known to be involved in the transport between the endoplasmic reticulum and Golgi apparatus [43] while OLA1 might regulate mRNA translation [44].

**Figure 6:**
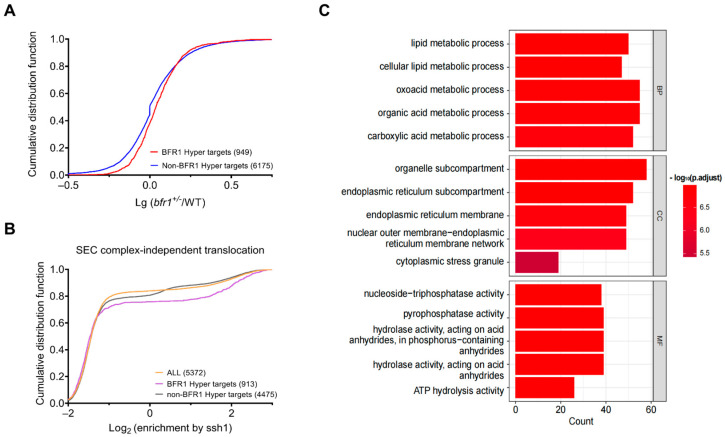
BFR1-HyperTRIBE targets are highly enriched in metabolic processes and membranous organelle-related pathways. (**A**) Cumulative distributions of log_10_ mRNA-abundance-ratio of *bfr1* heterozygote yeast (*bfr1^+/−^*) to its wild-type counterpart (WT) from BFR1-HyperTRIBE target group (red line, *n* = 949) and non-target group (blue line, *n* = 6175). Two-tailed, unpaired *t* test with Welch’s correction, *p* < 0.0001. (**B**) BFR1-HyperTRIBE targets are enriched with mRNAs translated on the endoplasmic reticulum (ER). Cumulative distributions are plotted for the groups of BFR1-HyperTRIBE targets (purple line, *n* = 913), non-targets (gray line, *n* = 4475) and all expressed mRNAs (FPKM ≧ 2, yellow line, *n* = 5372). The x-axis indicates the enrichment for mRNAs bound by ribosomes on the ER at the SSH1 translocon complex (log_2_(ssh1.heh2.7mchx enrichment)) obtained from published experiments [46]. (**C**) The GO term analysis results of biological process (BP), cellular component (CC) and molecular function (MF) are shown.

We analyzed the RNA-Seq results of the *bfr1*-heterozygous mutant (*bfr1^+/^^−^*) strain and wild type (WT) strain from NCBI, and obtained 1451 differentially expressed genes (FPKM_avg_ ≥ 2 and FPKM ratio (*bfr1^+/^^−^*/WT) multiples greater than 1.5) [45]. Furthermore, 36 overlapping genes were discovered between the 1678 BFR1-HyperTRIBE target genes (r10e10) and the differentially expressed genes between *bfr1^+/^^−^* and WT from RNA-Seq data (Figure 5E). The *bfr1^+/^^−^* strain had a lower BFR1 mRNA level compared with the wild-type strain (Figure 5F). Thus, the partial depletion of BFR1 resulted in the mRNA level change of the 36 overlapping genes, which included 28 up-regulated and 8 down-regulated genes (Figure 5G). Cumulative frequency distributions of the mRNA abundance changes after partial depletion of BFR1 were compared between two groups (BFR1-TRIBE target group and non-target group) (Figure 6A). The result showed a slight but significant difference between the two groups, suggesting that BFR1 mainly destabilizes the target mRNAs (Figure 6A). These observations were consistent with the reported research that BFR1 could trigger mRNA degradation under stress conditions like starvation [39].

In addition, BFR1 is known to be localized to the endoplasmic reticulum (ER) [25,37] so we examined whether BFR1 targets were enriched with ER-translated mRNAs, which were observed by the proximity-specific ribosome profiling strategy in yeast [46]. The cumulative frequency distributions of mRNA enrichment on the ER membrane showed that BFR1-hyperTRIBE targets were enriched with ER-translated mRNAs in comparison to all mRNAs or non-targets, especially those involved in Ssh1 translocon-mediated ER translocation events were significantly enriched (Figure 6B and Figure S5). The Ssh1 translocon on the ER membrane is thought to be associated only with signal recognition particle (SRP) receptor (SR) which mediates SR-dependent co-translational translocation of secretory proteins and membrane proteins in yeast (Figure 6B). Instead, the translocon accessory factor Sec63, which interacts with the Sec61 translocon, can mediate both co-translational and post-translational translocation (Figure S5B) [46]. Therefore, BFR1-TRIBE target mRNAs were mostly related to SR-dependent co-translational translocation. To determine the consensus sequence in the target mRNAs, we conducted STREME motif analysis either with ±100 bp regions or with ±200 bp regions from the HyperTRIBE edited sites. The analysis showed that there was no significantly enriched motif (all *p*-value > 0.1). To date, no evidence has indicated that BFR1 can directly bind to RNA; thus, we speculate that indirect association of BFR1 on RNA makes motif identification difficult and/or its binding on RNA does not require a strict consensus motif. GO term analysis of BFR1 targets revealed that most of its targets were involved in metabolic processes, cytoplasmic stress granules and the membranous organelle-related pathways (typically involving the ER membrane) (Figure 6C), which is in agreement with our data (Figure 6B) and a previous report [38].

## 3. Discussion

Our KHD1 and BFR1 HyperTRIBE data are consistent with these RBPs’ functions published in previous research. The roles of the KHD1 HyperTRIBE targets are enriched in the processes of filamentous growth, cell growth and adhesion (Figure 3C); thus, these targets are likely responsible for the phenotype of *khd1*-knockout yeast, such as increased filamentous growth, improved cell adhesion both between cells and to the plate [30]. Additionally, BFR1 HyperTRIBE targets are enriched with mRNAs translated on the ER (Figure 6B,C) and BFR1 mainly destabilizes its targets (Figure 6A).

As master regulators in cells, RBPs take part in gene regulation at multiple levels mostly by specifically recognizing RNA. To reveal the underlying mechanisms of RBP–RNA interactions, the profiling methods of this interaction network have been rapidly advancing. These methods each hold their own advantages. HITS-CLIP and its variants can detect the RNA targets from endogenous RBPs with relatively accurate binding site information. Instead, TRIBE, RNA tagging and STAMP are antibody-free methods, avoiding the problems associated with antibody specificity. These three methods also avoid the low UV-crosslinking efficiency problem, making them applicable in cells with cell wall structures, such as plants, fungi and bacteria. Of the three, TRIBE and STAMP require a lower number of starting cells and undergo standard RNA-seq library preparation protocol, making them easier for lab execution and accessible for single-cell level research. In addition, the research on a complex comprising multiple RBPs will benefit from combining different techniques in the same organism. Specifically, different RBPs, fused with different RNA modification enzymes, can be expressed in the same cells to mark the common and unique RBP targets. Thus, the development of various strategies for profiling RBP–RNA interactions will greatly contribute to research in the field of RNA biology.

The fusion protein-based system is a cornerstone of RBP target identification methodologies. The key challenge for establishing this kind of method in an untested species is obtaining an RNA modification enzyme with high activity in that particular species. The development of TRIBE in *Drosophila* used the fusion protein of *Drosophila* ADARcd (dADARcd) with an RBP [6], while the improved version HyperTRIBE employs a hyper-active dADARcd (E488Q) [13,14,15]. In mammalian HyperTRIBE, hADAR2cd (E488Q) was used to construct fusion proteins due to the higher editing efficiency of hADAR2cd (E488Q) compared to dADARcd (E488Q) in mammalian cells [13]. To adapt HyperTRIBE for application in yeast, we used the hyper-active catalytic domain from dADAR (E488Q), human ADAR2 (E488Q), and human ADAR2 (E488Q, V493T, N597K) [40] to construct HyperTRIBE fusion proteins. The HyperTRIBE with dADAR (E488Q) or hADAR2 (E488Q, V493T, N597K) failed to produce results with consistently high signal-to-noise ratios in yeast (Figure S6). However, HyperTRIBE with hADAR2 (E488Q) generated a high signal-to-noise ratio, great reproducibility and remarkable consistency with published data of tested RBPs. Our work is pioneering in terms of establishing a straightforward and practical HyperTRIBE method optimized for yeast. Furthermore, it provides support for other methodologies, which will be beneficial to the field in general. 

## 4. Materials and Methods

### 4.1. Plasmid Construction

RBPs of interest were cloned into pcDNA3-3HA-hADAR2cd-E488Q [13] to build pcDNA3-3HA-RBP-hADAR2cd-E488Q using the Gibson assembly method [47] (EasyGeno Single Assembly Cloning kit, Tiangen, Beijng, China). PGK1 and CYC1 were acquired from yeast gDNA. PGK1, 3HA-RBP-hADAR2cd-E488Q and CYC1 were cloned into pRS313 to obtain pRS313-PGK1-3HA-RBP-hADAR2cd-E488Q-CYC1. The V493T/N597K mutations were introduced into hADAR2cd-E488Q using the Fast Site-Directed Mutagenesis Kit (Tiangen, Beijng, China). The Khd1 was substituted with Myc tag to produce the pRS313-PGK1-hADAR2cd-E488Q-CYC1 plasmid. PGK1 was replaced by the PCR product of GAL1 to produce inducible plasmids. All cloned sequences were verified by Sanger sequencing (Genewiz, Jiangsu, China).

### 4.2. Yeast Cell Cultures for TRIBE Experiments

For the plasmids with a PGK1 promoter, the transfected cells were grown in SD/-His media (Coolaber, Beijing, China) for 12~16 h. For the plasmids with a GAL1 promoter, the selected cells were maintained in SD/-His first, then saturated cultures were seeded into SD/-His media at OD_600_ < 0.2. When the OD_600_ was close to 1, uninduced control cells were collected for Western blotting (WB). Glucose was eliminated by resuspending yeasts three times in SC/-His media (without dextrose) as it could inhibit GAL1-initiated transcription [48]. The incubation was continued in SG/-His (with 2% galactose, Aladdin, Shanghai, China) media at OD_600_ = 1 for several hours. The expression of TRIBE constructs was examined by WB and the verified samples were used for RNA preparation.

### 4.3. HyperTRIBE RNA-Seq Library Preparation and Analysis

The HyperTRIBE RNA-sequencing library was prepared followed the manufacturer’s instructions from the Illumina Stranded mRNA Prep kit in which dUTP was included during second strand cDNA synthesis (Hieff NGS^®^ Ultima Dual-mode mRNA Library Prep Kit for Illumina^®^, Yeasen, Shanghai, China). Hieff NGS^®^ Complete Adapter Kit for Illumina^®^ (Yeasen, Shanghai, China) was used for sample pooling. The quality of the NGS library was monitored using the Qubit dsDNA 915 kit (Agilent, Beijing, China) for Fragment Analyzer^TM^ (Agilent 5400, Beijing, China) [49] and qPCR (quantification of adaptor-added fragment). The qualified samples were sequenced by a paired-end–sequencing strategy (PE150, Novogene, Beijing, China) applying the NovaSeqTM6000 v1.5 reagent kit on an Illumina NovaSeq 6000 system (Illumina, San Diego, CA, USA). Two independent replicates of HyperTRIBE, KHD1-HyperTRIBE and BFR1-HyperTRIBE experiments were carried out. 

The raw data were processed according to a published protocol [14] with modification. Briefly, Cutadapt version 4.1 (https://cutadapt.readthedocs.io/en/stable/, accessed on 18 April 2023) was used for adaptor dislodgment. Trimmomatic (0.38) (http://www.usadellab.org/cms/?page=trimmomatic, accessed on 18 April 2023) was changed to the paired-end sequencing model for inferior read filtering. The clean data were aligned to the *Saccharomyces cerevisiae* genome (version R64-1-1) from SGD by STAR2.5.3a (https://github.com/alexdobin/STAR, accessed on 18 April 2023) (--outFilterMismatchNoverLmax 0.10 --outFilterMatchNmin 16 --outFilterMultimapNmax 1), while alignment index was generated from Saccharomyces_cerevisiae.R64-1-1.dna.toplevel.fa and Saccharomyces_cerevisiae.R64-1-1.103.gtf. After removing PCR duplicates, editing events were identified with the indicated threshold of reads and editing percentages and single-nucleotide polymorphisms were avoided using the wtRNA–RNA approach.

### 4.4. Database

RNA IP-Chip data for KHD1 were from the Saccharomyces Genome Database (https://www.yeastgenome.org/reference/S000127887, accessed on 19 September 2008). KHD1 targets detected in CLIP-seq were obtained from the database of the POSTAR3 CLIPdb Module. The differentially expressed genes (DEG) of KHD1 were calculated from microarray data from the wild type strain S288C and *khd1*-knockout strain (*hek2Δ*) from NCBI GEO Sample GSM843507 (https://www.ncbi.nlm.nih.gov/geo/query/acc.cgi?acc=GSM843507, accessed on 6 December 2011). The RNA-tagging data for BFR1 were from SGD (https://www.yeastgenome.org/reference/S000182016, accessed on 2 November 2015), and the RIP-Chip data of BFR1 were from SGD (https://www.yeastgenome.org/reference/S000128183, accessed on 28 October 2008). The differentially expressed genes (DEG) of BFR1 were calculated by comparing RNA-Seq data from the wild type strain BY4743 with the BFR1 heterozygote mutant *BFR1/bfr1Δ::GFP* (*bfr1^+/−^*) obtained from NCBI GEO Sample GSM4240918 (https://www.ncbi.nlm.nih.gov/geo/query/acc.cgi?acc=GSM4240918, accessed on 21 February 2020).

### 4.5. Motif Analysis and GO Term Analysis

In the reference genome (*Saccharomyces_cerevisiae*. R64-1-1), the 100 bp upstream and 100 bp downstream regions of the edited sites were selected to carry out motif analysis. The same length (201 bp) and same number of background regions were randomly selected from non-target genes. The analysis was carried out for two biological replicates and two similar high-confidence motifs for KHD1 were observed each from the MEME algorithm [50] (https://meme-suite.org/meme/tools/meme, accessed on 18 April 2023, parameters: -discriminative mode -zoops mode -0-order model of sequence -minw 10 -maxw 15) and XSTREME algorithm [51] (https://meme-suite.org/meme/tools/xstreme, parameters: -Ray2013 S. cerevisiae database -minw 10 -maxw 15 -zoops mode).

GO enrichment was analyzed using the R Programming Language (clusterProfiler [52], http://www.bioconductor.org/packages/release/bioc/html/clusterProfiler.html, accessed on 18 April 2023). The ID type of GENENAME was converted to ENTREZID with function bitr loaded with org.Sc.sgd.db (yeast genome ID, https://www.yeastgenome.org/) before proceeding to use the enrichGO [52] function. GO analyses of HyperTRIBE target genes were analyzed against a background of a similar number of transcripts that were randomly selected from org.Sc.sgd.db.

## Data Availability

All data needed to evaluate the conclusions in the paper are present in the paper and/or the Supplementary Materials. Raw and processed next-generation sequencing data from this study were deposited at the National Center for Biotechnology Information Gene Expression Omnibus database with accession number PRJNA948539. Additional data related to this paper may be requested from the authors.

## Supplementary Materials

The following supporting information can be downloaded at: https://www.mdpi.com/article/10.3390/ijms24109033/s1. Refs. [25,27,28,29,33,37,39,42,46,52,53] are cited in Supplementary Materials.
