# Supplementary material for: Identification of RNA-Binding Protein Targets with HyperTRIBE in Saccharomyces cerevisiae
Source: Int J Mol Sci. 2023 May 20;24(10):9033. doi: 10.3390/ijms24109033 (PMC10218906; doi:10.3390/ijms24109033)
Supplement: Supplementary file 1 [file ijms-24-09033-s001.zip › ijms-2381896-Supplementary figures and experimental procedures.pdf]

Supplemental Figures

A

Protein name alias	Domain organization	Mw AAs	Function	Yeast mRNA interactomes RBD	Previously known RNA target
KHD1 HEK2,YBL032W, YBL0418	KH_1 KH_1 KH_1	42 kDa 381 AA	Localization	Classical RBD	ASH1、MID2、SCW11、SLG1
BFR1 YOR198C	Smc	55 kDa 470 AA	Translation	Unknown RBD	VNX1、TDP1

B

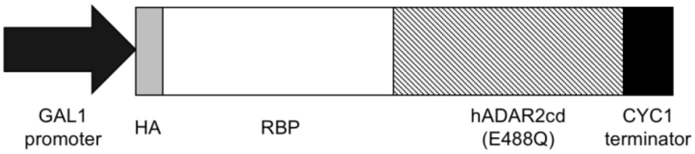

C

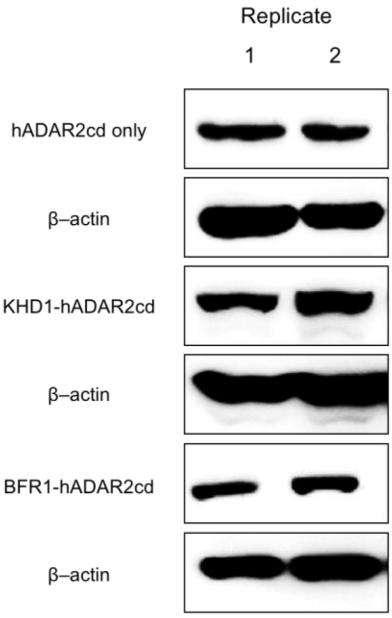

Supplementary Figure S1. The HyperTRIBE fusion protein expression is confirmed by western blotting. (A) Information about yeast RNA-binding protein KHD1 and BFR1. KHD1 protein has three KH RNA-binding motifs, and has been confirmed to bind ASH1 mRNA and be necessary for the effective localization of target mRNA[33]. BFR1 lacks typical RNA binding domains and participates in the protein secretion pathway[25, 37]. It is located in the endoplasmic reticulum (ER) under normal conditions and in P-body after

stress[39]. **(B)** The map of DNA construct to express the HyperTRIBE fusion protein. GAL1 is a galactose-inducible promoter, and hADAR2cd (E488Q) is the catalytic domain of human ADAR2 with a point mutation E488Q. **(C)** Western blot analysis shows the expression of HyperTRIBE fusion proteins in yeast. The hADAR2cd, KHD1-hADAR2cd, and BFR1-hADAR2cd all with E488Q were detected using the antibody against HA-tag. β-actin was used as a loading control.

A

KHD1		
5'UTR	CDS	3'UTR
0	1443	6

B

BFR1		
5'UTR	CDS	3'UTR
0	1647	13

C

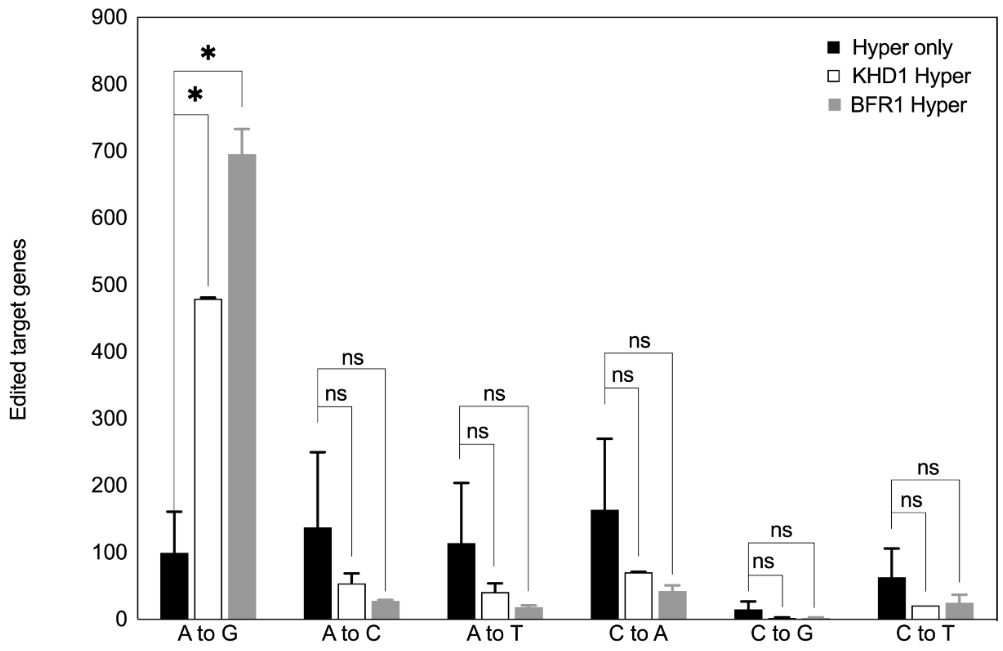

Supplementary Figure S2. The number of edited genes with different types of sequence alterations. (A, B) The editing sites of KHD1-HyperTRIBE (A) and BFR1-HyperTRIBE (B) are mainly located in the protein coding region. **(C)** The different types of mRNA sequence alteration events are counted in HyperTRIBE-expressed cells using mRNA sequence from wild type cells as reference. Only A-to-G edits are significantly frequent in KHD1-HyperTRIBE and BFR1-HyperTRIBE samples compared with Hyper-only samples. Other kinds of edits are not significantly enriched when comparing RBP-HyperTRIBE with Hyper-only. *N*=2, r20e10, **P*=0.05, paired one-tailed Student’s t test.

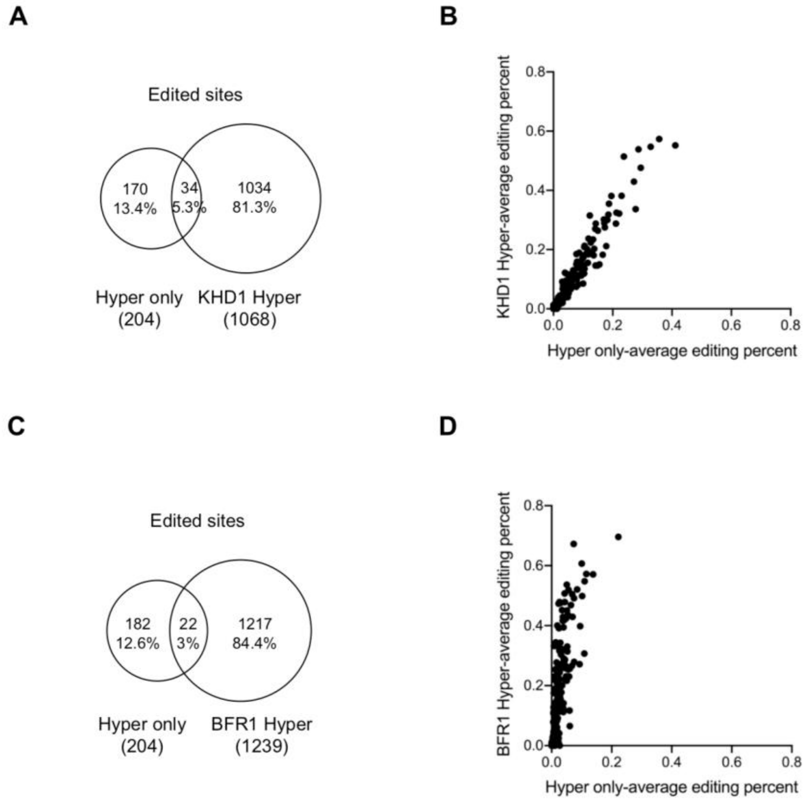

Supplementary Figure S3. A small proportion of editing sites are overlapped between RBP-HyperTRIBE and Hyper-only. (A) The editing sites identified in KHD1-HyperTRIBE are much more than those in Hyper-only. About 5.3% of editing sites are overlapped between Hyper-only and KHD1-HyperTRIBE (Read≥20, editing≥10%) as showed in Venn diagram. The editing sites detected in two biological replicates are presented. **(B)** Editing percentages in KHD1-HyperTRIBE are higher than those in Hyper-only for the common editing sites. The editing percentages are averaged over two biological replicates. **(C)** BFR1-HyperTRIBE generates much more editing sites than Hyper-only, and ~3% of editing sites are overlapped between Hyper-only and BFR1-HyperTRIBE (Read≥20, editing≥10%). The editing sites detected in two biological replicates are presented. **(D)** A much higher editing percentage is detected in BFR1-HyperTRIBE compared with that in Hyper-only for the common sites. The editing percentages are averaged over two biological replicates.

Gene	RNA enriched in bud tip	Protein enriched in bud tip	Protein levels in KHD1 OE cells	RIP-Chip with KDH1 antibody	The colocalization of mRNA with KHD1 protein	RNA pulldown
ASH1†	Yes	Yes	Down	✓	✓	✓
EGT2	Partial	Yes	-	✓	✓	-
IST2	Yes	Yes	ND	✓	✓	-
WSC2	Yes	Yes	ND	✓	✓	-
TAM41	Yes	No	-	X	X	-
IRC8	Yes	Yes	-	X*	-	-
MTL1	Weak	ND	UP	✓	✓	✓

Supplementary Figure S4. KHD1-HyperTRIBE identified 7 bud tip localization mRNAs as KHD1 targets. † In addition to ASH1, the other six transcripts are bud tip localization mRNAs detected by KHD1-HyperTRIBE. The table summarizes the reported information about 7 bud tip localization mRNAs[27, 29]. Yes, 90% bud localization; Partial, 50–60% localization; Weak, 15–30% localization; No, unlocalized (5% localization); ND, not determined; -, not assayed. * IRC8 was not enriched in RIP[28] but enriched in another set of RIP (q<0.05)[42].

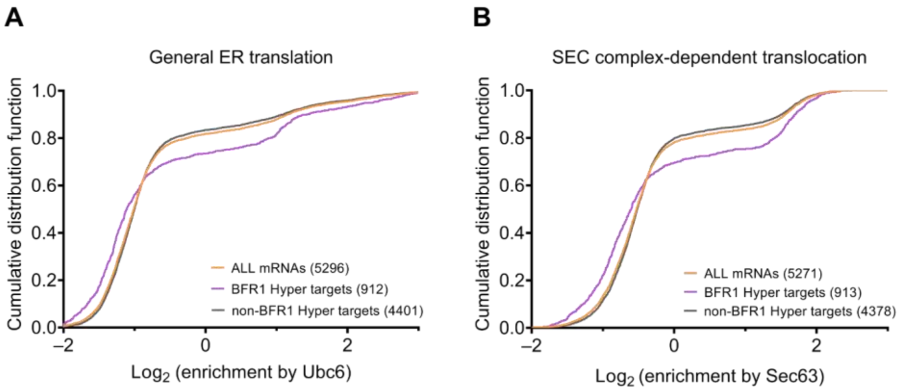

Supplementary Figure S5. BFR1 targets are enriched with mRNAs translated on ER. Cumulative distributions are plotted for the groups of BFR1-HyperTRIBE targets, non-targets, and all expressed mRNAs (FPKM≧2) for the following attributes: enrichment for mRNAs bound by ribosomes at the ER ($\log_2$(ubc6.7mchx enrichment)) **(A)**, or at the SEC complex ($\log_2$(sec63.7mchx enrichment)) **(B)**, obtained from the published ER-specific ribosome profiling experiments[46].

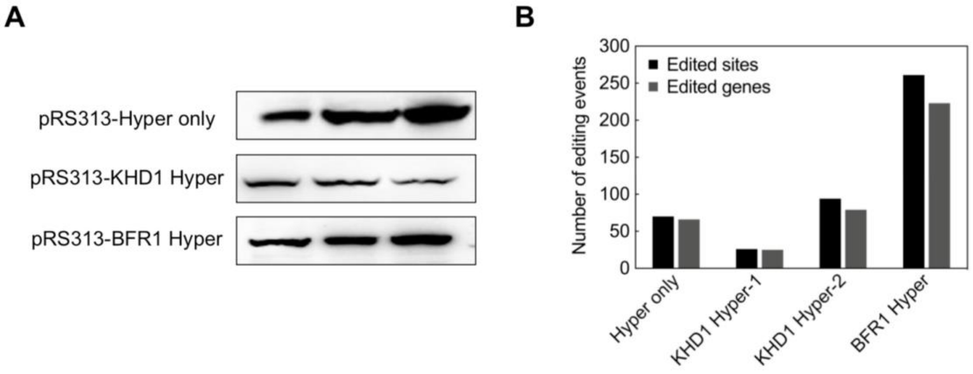

Supplementary Figure S6. HyperTRIBE using hADAR2cd with three point mutations (E488Q, V493T, N597K) doesn't work consistently. (A) The protein expression of Hyper-only, KHD1-Hyper, and BFR1-Hyper all with three point mutations are confirmed by western blotting. **(B)** The bar graph shows the numbers of edited sites and edited genes from HyperTRIBE conducted by hADAR2cd with three point mutations.

Supplemental experimental procedures

Yeast transformation

All *Saccharomyces cerevisiae* strains were generated from BY4742 (*MATα; his3Δ1; leu2Δ0; lys2Δ0; ura3Δ0*). The transformation method was adapted from prior protocol[52]. Centrifugation was completed for 5 min at 3000 rcf at room temperature (RT) unless otherwise specified (Eppendorf, Hamburg, Germany). PEG 3350 and LiAc were autoclaved prior to use. A fresh streaked colony of BY4742 was inoculated in a 2-5 mL YPD medium (yeast extract, peptone, 2% autoclaved and isolated dextrose) at 30°C and 240 rpm for 12-16 h. Then, cultured yeasts were added into a fresh YPD medium at OD_{600} ~0.2, and the mixture was shaked until OD_{600} reached 0.6-0.7. The Pellet from 1.25 mL cultivation was washed once with 1 mL sterile water then immersed in 100 μL 1 M LiAc for 5 min at RT. The supernatant was removed by centrifuging for 3 min at 3000 rcf at RT. Next, the following reagents were added to the pellet sequentially: sterile water, 33% PEG 3350 (w/v, mixed by pipetting immediately after adding, solarbio, Beijing, China), 100 mM LiAc (QualitYard, Beijing, China), 0.28 mg/mL salmon sperm DNA (pretreatment with 95°C heating for 5 min and instant cooling on ice, Solarbio, Beijing, China), and 0.5-2 μg plasmid to a total volume of 360 μL, then blended gently for 1 min. The compound was incubated at 30°C for 30 min and heat shocked at 42°C (for BY4742) for 20-30 min. The pellet was collected after centrifuging for 3 min at RT. 1 mL YPD was added for growing another 2 h at 30°C and 150 rpm. Cells were cleaned with sterilized water twice. The resuspended cells were plated on SD/-His (2% dextrose) agar and cultured for 2-4 days.

Purification of gDNA and RNA

Yeast cells were centrifugated at 6,000 rcf at 4°C. Cordless motor and pellet pestles (Thermo Fisher Scientific™, Walthum, MA, USA) were utilized for cell homogenization in

100-200 uL of TRIzol^{TM} reagent (Thermo Fisher Scientific™, Walthum, MA, USA). Grinding for 30 s and chilling on ice for 30 s were repeated five times for cell homogenization. Then, TRIzol^{TM} reagent was further added to final volume of 1 mL per 50-70 µL of yeast pellet. Total RNA purification was followed the manufacturer's instructions.

Extraction of gDNA was carried out in agreement with *Molecular Cloning: A Laboratory Manual [53]*. Acid-washed beads (Solarbio, Beijing, China) were prepared by steeping overnight in acid solution (6M HCl:H_2O=1:50). After washing with ultrapure water (Merck, Darmstadt, Germany) ten times, beads were autoclaved and dried for later use.
